# Deep enteroscopy in children: techniques, applications, and future directions

**DOI:** 10.3389/fped.2025.1562075

**Published:** 2025-03-12

**Authors:** Brett J. Hoskins

**Affiliations:** Division of Pediatric Gastroenterology, Hepatology, and Nutrition, Department of Pediatrics, Indiana University School of Medicine, Riley Hospital for Children at IU Health, Indianapolis, IN, United States

**Keywords:** pediatric deep enteroscopy, balloon-assisted enteroscopy, small bowel disorders, diagnostics and therapeutics, pediatric gastroenterology, push enteroscopy, single-balloon enteroscopy, double-balloon enteroscopy

## Abstract

Deep enteroscopy, encompassing push enteroscopy (PE) and balloon-assisted enteroscopy (BAE), has revolutionized the diagnosis and treatment of pediatric small bowel disorders. This review examines the evolving role of these techniques in managing conditions such as obscure gastrointestinal bleeding, Crohn's disease, polyposis syndromes, strictures, and small bowel tumors. While PE is effective for both diagnostic and therapeutic interventions in the proximal small bowel, its limited insertion depth has driven the adoption of BAE techniques. These include single-balloon enteroscopy (SBE) and double-balloon enteroscopy (DBE), which provide deeper and more comprehensive access. Both BAE modalities offer greater insertion depth and stability, enabling advanced therapeutic interventions such as polypectomy, stricture dilation, and hemostasis. Pediatric-specific data demonstrate high diagnostic yields for BAE, with comparable outcomes between SBE and DBE. These techniques have proven safe across diverse indications, though younger children may experience slightly higher complication rates due to anatomical considerations. Despite these advancements, challenges persist, including a limited evidence base in pediatrics, barriers to training, and the need for standardized protocols. Additionally, emerging innovations such as artificial intelligence offer opportunities to enhance diagnostic accuracy and procedural efficiency. Comparative analyses of PE, BAE, and capsule endoscopy are necessary to refine procedural selection and optimize outcomes in pediatric patients. Furthermore, structured pediatric training programs and simulation-based learning could address competency gaps, ensuring safe and effective application of these techniques. By addressing current research gaps, embracing technological advancements, and tailoring approaches to pediatric populations, deep enteroscopy can continue to transform the management of small bowel disorders in children.

## Introduction

Deep enteroscopy encompasses push enteroscopy (PE) and balloon-assisted enteroscopy (BAE), which includes single-balloon enteroscopy (SBE) and double-balloon enteroscopy (DBE). These advanced techniques have transformed the diagnostic and therapeutic management of pediatric small bowel disorders, allowing for interventions that were previously limited to surgical approaches (1–5). While BAE enables deeper and more comprehensive access to the small intestine, its complexity and technical demands necessitate specialized expertise and training. Consequently, its availability is largely limited to high-volume centers, posing challenges for widespread implementation in pediatric gastroenterology. Additionally, the small intestine's intricate anatomy and unique physiological characteristics in children necessitate a meticulous and tailored approach to ensure procedural success (2, 6, 7).

The development of capsule endoscopy, a minimally-invasive imaging modality, served as a catalyst for deep enteroscopy by highlighting the need for direct visualization and intervention (2, 4, 8–10). Today, deep enteroscopy is an indispensable tool in the management of pediatric conditions such as obscure gastrointestinal bleeding (OGIB), polyposis syndromes, Crohn's disease, strictures, Meckel's diverticula, and small bowel tumors. Beyond diagnostics, it facilitates therapeutic interventions, including polypectomy, hemostasis, stricture dilation, foreign body retrieval, defect closure, and even endoscopic cholangioscopy in children with altered anatomy (1–4, 6, 7, 9–20).

Although PE provided early capabilities for small bowel evaluation (2, 9, 21), its limited insertion depth led to the development of balloon-assisted techniques like SBE and DBE. These methods achieve deeper insertion and greater therapeutic stability using overtubes with inflatable balloons to pleat the small bowel onto the endoscope (3, 6, 22). Depending on the target area within the small intestine, procedures can be performed anterograde (oral) or retrograde (rectal) (1, 6). While spiral enteroscopy (SE) is effective in adults, its application in pediatrics remains limited due to equipment constraints and sparse data (6).

This review examined the pediatric literature on deep enteroscopy, analyzing its indications, methodologies, outcomes, and challenges while highlighting its evolving role in small bowel disease management. Tables 1–3 provide detailed comparisons of PE, SBE, and/or DBE, outlining their respective insertion methods, equipment specifications, depth of insertion, diagnostic yields, therapeutic applications, and procedural limitations. This comparative framework lays the foundation for the subsequent in-depth exploration of each modality.

**Table 1:** Comparison of push enteroscopy, single-balloon enteroscopy, and double-balloon enteroscopy in children.

Feature	Push enteroscopy	Single-balloon enteroscopy	Double-balloon enteroscopy
Insertion method	Push-and-pull using a flexible scope advanced manually	Single balloon on overtube to anchor and pleat the small bowel	Dual balloons, one on scope and one on overtube, to anchor and pleat the small bowel
Enteroscope diagram	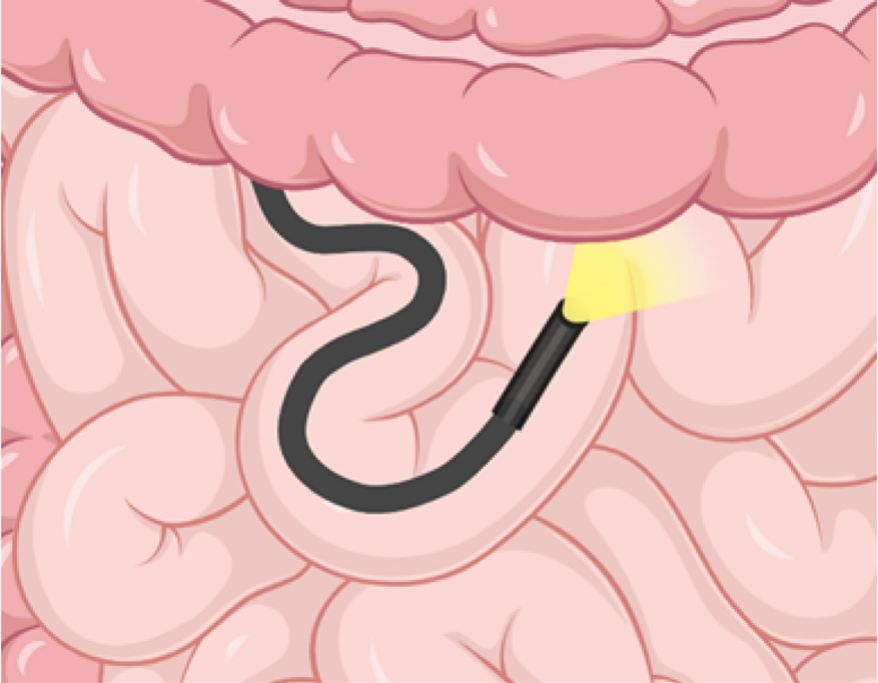	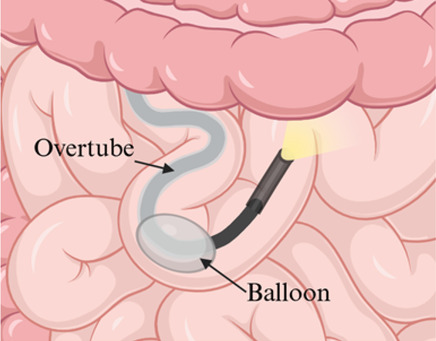	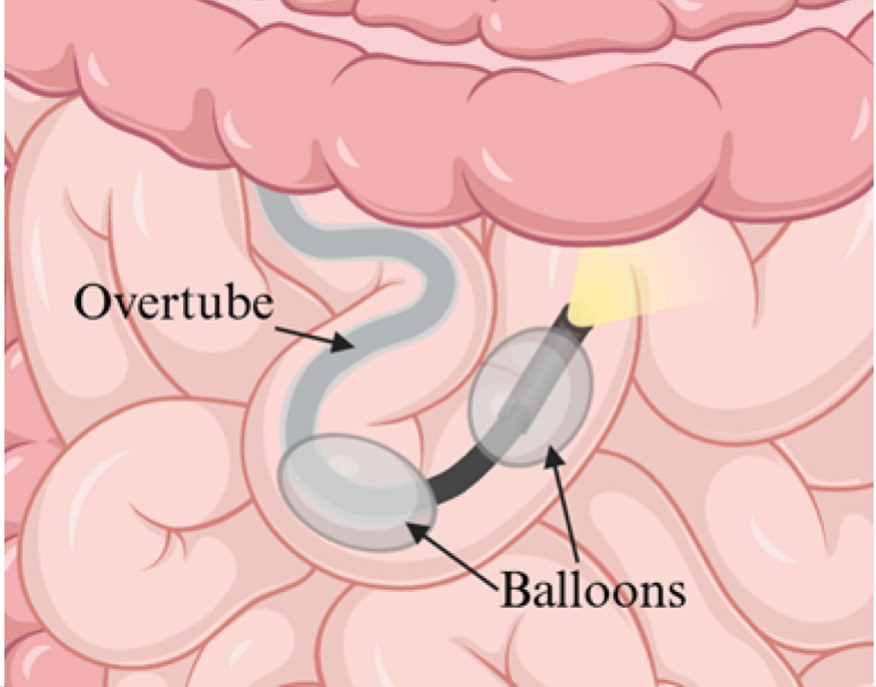
Equipment used	Push enteroscope or colonoscope (pediatric or adult) Optional single-use overtube	Balloon enteroscope Single-use overtube Balloon control unit	
Endoscope specifics^a^	Working length: 150–250 cm Outer diameter: 9.2–11.7 mm Channel diameter: 2.8–3.8 mm	Working length: 152–200 cm Outer diameter: 9.2 mm Channel diameter: 2.8–3.2 mm	Working length: 155–200 cm Outer diameter: 7.5–9.4 mm Channel diameter: 2.2–3.2 mm
Overtube specifics	Overtube optional	Outer diameter: 13.2 mm Material: silicone	Outer diameter: 11.2–13.2 mm Material: silicone or latex
Minimal age and weight^b^	Age: ≥2 years Weight: ≥10 kg	Age: ≥3 years Weight: ≥13.5 kg	Age: ≥2 years Weight: ≥12 kg
Depth of insertion	45–90 cm past ligament of Treitz Up to 132 cm beyond pylorus	Mean depth up to 258 cm	Mean depth up to 253 cm
Reachable areas	Proximal small bowel (anterograde) Distal small bowel (retrograde)	Proximal and mid-to-distal small bowel	
Reported uses (most common)	Crohn's disease, GI bleeding, polyposis syndromes, abdominal pain, chronic diarrhea	Chronic abdominal pain, GI bleeding, diarrhea, Crohn's disease, polyposis syndromes	GI bleeding, polyposis syndromes, abdominal pain, Crohn's disease
Diagnostic yield	24%–84%	47%–74%	58.8%–78.6%
Therapeutic capabilities	Similar among modalities (e.g., hemostasis, polypectomy, stricture dilation, foreign body removal, etc.)		
Advantages	Minimally invasive Widely available Lower cost Effective for proximal small bowel	Allows for deeper evaluation of the small bowel compared to PE Improved therapeutic stability	Allows for deeper evaluation of the small bowel compared to PE Improved therapeutic stability (may be enhanced compared to SBE)
Limitations	Limited insertion depth Difficult distal access Limited pediatric-specific data	Requires specialized equipment Patient size constraints Limited pediatric-specific data	Requires specialized equipment Patient size constraints
Common adverse events	Mild abdominal pain and discomfort (infrequent)		
Serious adverse events	Bleeding and perforation (<1% in adult data)	Post-polypectomy bleeding Post-polypectomy perforation (0%–1.8% in pediatric data)	Post-polypectomy bleeding Pancreatitis (0%–5.4% in pediatric data)
Training recommendations	Clinical experience and simulation recommended by ASGE and ESGE	Clinical experience and simulation recommended ASGE: ≥10 upper deep enteroscopy cases and 20 retrograde DBE cases ESGE: ≥75 DAE procedures with ≥35 retrograde cases	

ASGE, American Society for Gastrointestinal Endoscopy; BAE, balloon-assisted enteroscopy; DBE, double-balloon enteroscopy; ESGE, European Society for Gastrointestinal Endoscopy; PE, push enteroscopy; SBE, single-balloon enteroscopy.

Image Credit: BioRender. Hoskins, BJ. (2025). https://biorender.com/q62l993.

^a^ Equipment specifics for PE vary depending on endoscope chosen. SBE and DBE data reported are based on the Olympus SBE and Fujifilm DBE systems.

^b^ Based on available pediatric data.

**Table 2:** Summary of balloon-assisted enteroscopy studies in children.

Article	Procedure	Number of cases	Age Range (years)	Indications	Main findings	Serious complications
Barth and Channabasappa (52)	SBE	7 children 7 procedures	5–17	Abdominal pain, anemia, diarrhea, GI bleeding, polyposis	SBE was feasible and safe in initial experience	None
Belsha et al. (47)	DBE (1 DBE-laparoscopy)	16 children 22 procedures	8–16	PJS	Successful clearance of large small bowel polyps in all patients	Post-DBE-laparoscopy pelvic abscess (1)
Bizzarri et al. (46)	SBE	10 children 23 procedures	5.6–15.6	PJS	SBE is effective for polyp management	Post-polypectomy perforation (1)
Chen et al. (48)	DBE	61 children 72 procedures	6–14	Abdominal pain, occult GI bleeding	Diagnostic yield: 77.5% (mostly, non-specific enteritis or Crohn's disease)	None
Di Nardo et al. (12)	SBE	30 children 36 procedures	7–18	Crohn's disease (suspected or established)	SBE was useful for diagnosing and managing Crohn's disease with a high therapeutic yield	None
Gurkan et al. (19)	DBE	5 children^a^ 5 procedures	10–12	Abdominal pain, anemia, diarrhea, PJS	DBE was safe	None
Hagiwara et al. (4)	SBE and DBE (some DBE-cholangioscopy)	79 children 96 procedures	1–17	Abdominal pain, Crohn's disease, diarrhea, GI bleeding, polyposis	Diagnostic yield: 48% Procedure duration of oral-route was longer than anal-route	Post-polypectomy bleeding (1); Post-DBE-cholangioscopy pancreatitis (1)
Li et al. (14)	SBE and DBE	41 children 82 procedures	5–14	PJS	SBE was safe and effective for PJS polypectomy	Post-polypectomy perforation (1 case)
Lin and Erdman (43)	DBE	11 children 13 procedures	8–20	Abdominal pain, anemia, diarrhea, GI bleeding, polyposis	Diagnostic yield: 46% DBE was safe	None
Matsushita et al. (67)	DBE	40 children 62 procedures	3–18	Abdominal pain, Crohn's disease, OGIB, PJS (post-operative vs. nonoperative patients)	Insertion may be more difficult, but DBE was safe post-operatively	None
Nishimura et al. (7)	DBE (some DBE-cholangioscopy)	48 children 92 procedures	4–18	Abdominal pain, biliary stricture post-liver transplant, OGIB, polyposis	Diagnostic yield: 65% Successful endoscopic therapy in 56% of biliary stricture cases	Post-polypectomy bleeding (1)
Reddy et al. (5)	SBE	174 children 189 procedures	3–18	Abdominal pain, diarrhea, GI bleeding, vomiting	Diagnostic yield: 67.2% (mostly, ileal and jejunal ulcers) SBE was safe and effective	None
Shen et al. (44)	DBE	30 children 35 procedures	6–17	Abdominal pain, diarrhea, OGIB	Diagnostic yield: 96.7% Management altered in 90% of cases DBE was feasible and safe with high therapeutic impact	None
Thomson et al. (1)	DBE	14 children 14 procedures	8.1–16.7	Abdominal pain, OGIB, PJS	Diagnostic yield: 78.6% Therapeutic success in 64.3%	None
Uchida et al. (17)	DBE	67 children 106 procedures	3–19	Suspected Crohn's disease	Diagnostic yield: 88% DBE was safe and effective	None
Urs et al. (16)	DBE (some DBE-laparoscopy)	58 children 113 procedures	1–18	Abdominal pain, Crohn's disease, OGIB, PLE, polyposis	Diagnostic yield: 70.7% Therapeutic intervention in 46.5%	Anastomotic perforation (1) Post DBE-laparoscopy pelvic abscess (1)
Wu et al. (18)	DBE	37 children 42 procedures	4–16	Abdominal pain, diarrhea, GI bleeding	Diagnostic yield: 75.7% DBE improved diagnosis and management	None
Yokoyama et al. (15)	DBE (many DBE-cholangioscopy)	117 children 257 procedures	3–18	Abdominal pain, biliary stenosis or stones, Crohn's disease, OGIB, polyposis	Diagnostic yield: 58.8% Higher complication rate if <10 years of age (5.4 vs. 10.4%)^b^ DBE is safe and feasible	Post-polypectomy perforation (1) and bleeding (4) Post-DBE-cholangioscopy bile duct injury (2) and pancreatitis (3)
Zhu et al. (20)	DBE	10 children 10 procedures	3.3–12.1	Meckel's diverticular bleeding	Diagnostic yield: 100% DBE is safe and reliable for identifying Meckel's diverticula	None

DBE, double-balloon enteroscopy; GI, gastrointestinal; OGIB, obscure gastrointestinal bleeding; PJS, Peutz-Jeghers syndrome; PLE, protein-losing enteropathy; SBE, single-balloon enteroscopy.

^a^ Adult cases excluded.

^b^ Includes DBE-cholangioscopy cases.

**Table 3:** Equipment specifications for balloon-assisted enteroscopes.

Enteroscope or overtube model	System	Working length (mm)	Outer diameter (mm)	Channel size (mm) or material	Associated overtube or enteroscope
Balloon-assisted enteroscopes					
Fujifilm (Fujinon)					
EC-450BI5	DBE	1,820	9.4	2.8	TS-13101
EN-450P5/20	DBE	2,000	8.5	2.2	TS-1214B, TS-12140
EN-450T5	DBE	2,000	9.4	2.8	TS-1314B, TS-13140
^a^EN-580T	DBE	2,000	9.4	3.2	TS-13140
^a^EI-580BT (Short)	DBE	1,550	9.4	3.2	TS-1314B, TS-13101
EN-580XP (Slim)	DBE	2,000	7.5	2.2	TS-1114B
^a^EN-840T	DBE	2,000	9.4	3.2	TS-1314B
Olympus					
SIF-H190	SBE	2,000	9.2	3.2	ST-SB1
SIF-H290S (Short)	SBE	1,520	9.2	3.2	ST-SB1S
^a^SIF-Q180	SBE	2,000	9.2	2.8	ST-SB1
SIF-Q260	SBE	2,000	9.2	2.8	ST-SB1
Overtubes					
Fujifilm (Fujinon)					
TS-1114B	DBE	1,400	11.2	Silicone	EN-580XP
TS-1214B	DBE	1,400	12.2	Silicone	EN-450P5/20
^a^TS-1314B	DBE	1,400	13.2	Silicone	EN-450T5, EN-580T, EN-840T
TS-12140	DBE	1,450	12.2	Latex	EN-450P5/20
^a^TS-13140	DBE	1,450	13.2	Latex	EN-450T5, EN-580T
TS-13101	DBE	1,050	13.2	Latex	EC-450BI5, EI-580BT
Olympus					
^a^ST-SB1	SBE	1,320	13.2	Silicone	SIF-H190, SIF-Q180, SIF-Q260
ST-SB1S	SBE	880	13.2	Silicone	SIF-H290S

DBE, double-balloon enteroscopy; SBE, single-balloon enteroscopy.

^a^ Currently available in the United States.

## Push Enteroscopy (PE)

PE is a minimally-invasive technique that utilizes a flexible endoscope to access deeper sections of the small bowel via an advance-and-reduce (push-and-pull) method. It is commonly performed antegrade in the upper gastrointestinal tract for proximal small bowel conditions, but can also be performed retrograde via the anal route to access the distal small bowel.

### Equipment specifics

PE can be performed using either a dedicated enteroscope or a pediatric or adult colonoscope (23, 24). Several push enteroscope models are available from manufacturers such as Olympus, Fujinon, and Pentax, featuring working lengths of 150–250 cm, outer diameters of 9.2–11.7 mm, and working channel diameters of 2.8–3.8 mm (24, 25). Equipment specifics for pediatric and adult colonoscopes vary depending on the manufacturer, and can provide an additional 23–67 cm of working length compared to gastroscopes. Overtubes may be used to decrease gastric looping and enable deeper insertion (25).

### Clinical applications

PE is an effective diagnostic and therapeutic tool for managing proximal small bowel conditions in pediatric patients, including OGIB, ulcers, strictures, polyps, and lymphangiectasia (9, 11, 21, 23). It can help diagnose Crohn's disease, polyps, eosinophilic gastroenteritis, and other conditions, significantly influencing clinical management (21).

In adult studies, PE is used for evaluating abnormal imaging, localizing and treating lesions, sampling tumors, managing polyposis syndromes, retrieving foreign bodies, facilitating ERCP in postsurgical anatomy, placing jejunostomy tubes, and addressing chronic diarrhea and malabsorption (8, 26). The American Society for Gastrointestinal Endoscopy (ASGE) guidelines highlight its diagnostic and therapeutic utility for small bowel bleeding, with diagnostic yields ranging from 24%–56% (27). For OGIB specifically, PE identifies bleeding sources in the proximal small intestine and provides therapeutic options, improving outcomes while minimizing invasiveness (27, 28). Although pediatric-specific data are limited, adult studies report diagnostic yields ranging from 3%–70%, depending on insertion depth and lesion location (29). An early study by Darbari et al. showed a diagnostic yield of 84% in 44 children (21).

In pediatric studies, insertion depths of 132 cm beyond the pylorus were achieved using an SIF-190 enteroscope (Olympus America Inc., Center Valley, PA) in children as young as 2 years of age and weighing at least 10 kg (21). According to American College of Gastroenterology (ACG) guidelines, PE typically advances 45–90 cm beyond the ligament of Treitz (29).

PE is valuable in pediatric Crohn's disease, enabling biopsies and therapeutic stricture dilation (9, 11). It also facilitates polypectomy for syndromes like Peutz-Jeghers syndrome (PJS) and familial adenomatous polyposis, effectively removing duodenal/jejunal polyps and reducing surgical interventions (21). These findings position PE as a minimally invasive, versatile option for pediatric small bowel disorders.

### Safety and adverse events

PE is generally considered safe in pediatric patients, although data specific to this population remain limited. In a study by Darbari et al., PE was safely performed in 44 children (median age: 10 years) without any significant adverse events. Minor complications, such as transient abdominal pain and discomfort, were the most commonly reported issues (21).

While pediatric-specific data are sparse, adult studies offer additional insight into PE's safety profile. In adults, the overall complication rate is low, with major events like perforation and bleeding occurring in less than 1% of cases (30). Other potential complications include infection and sedation-related cardiopulmonary events. According to ACG guidelines, careful patient selection and procedural technique further minimize risks (29).

Although larger pediatric studies are needed, current evidence supports PE as a safe procedure for evaluating and managing proximal small bowel conditions in children, with a low incidence of significant complications.

### Training and implementation

Effective use of PE requires advanced training, though its learning curve is less steep compared to modalities with specialized equipment. Pediatric endoscopists typically gain proficiency through observation, mentorship, and simulation-based modules designed to replicate small bowel navigation techniques (31). The ASGE 2013 guidelines recommend training programs combining virtual and hands-on experience under expert supervision to enhance procedural skills and confidence (31). Similarly, the European Society of Gastrointestinal Endoscopy (ESGE) recommends a minimum of 75 procedures to achieve competence in device-assisted enteroscopy (DAE) (32), which can be particularly challenging in the pediatric population due to the fewer cases available.

While equipment costs can be challenging, using a colonoscope in certain cases helps reduce expenses. PE's ability to replace exploratory surgery and provide therapeutic interventions offers significant long-term healthcare savings (33, 34). Training also emphasizes careful patient selection, proper techniques, and effective complication management to ensure safety and optimize outcomes (35).

### Research gaps

While PE has been adopted in pediatrics, its use remains somewhat limited by the lack of robust pediatric-specific studies. One study demonstrated its safety and diagnostic utility in children (21), though larger multicenter trials are needed to fully evaluate its safety, efficacy, and long-term outcomes in this population. Furthermore, differences in anatomy, physiology, and disease manifestations between adults and children challenge the direct extrapolation of adult findings, highlighting the need for dedicated pediatric research.

## Balloon-Assisted Enteroscopy (BAE)

BAE uses an overtube equipped with either one or two inflatable balloons to achieve incremental advancement of the endoscope, allowing for deep intubation of the small bowel. This technique offers superior depth of insertion compared to traditional methods, making it a crucial tool for diagnosing and managing small bowel disorders in children. The two primary modalities, SBE and DBE, are widely utilized to address mid-to-distal small bowel pathologies. Although DBE was initially thought to achieve greater insertion depth, recent evidence demonstrates that the outcomes of SBE and DBE are comparable (36–38).

### Equipment specifics

The equipment used for BAE varies between SBE and DBE, each with specific features designed to optimize performance. SBE employs a single balloon on the overtube to anchor the bowel and pleat it over the endoscope. Models such as the Olympus SIF-H190 (Olympus America, Inc., Center Valley, PA, USA) feature a 9.2 mm outer diameter, a working length of 200 cm, and a 3.2 mm instrument channel, while the slightly older SIF-Q260 model offers similar specifications but with a smaller 2.8 mm instrument channel. Both models are compatible with the ST-SB1 splinting tube, available in silicone, which has an outer diameter of 13.2 mm and a working length of 132 cm (29, 39–41).

In contrast, DBE uses two balloons—one on the overtube and another on the endoscope itself—to facilitate a push-and-pull technique. Fujifilm (Valhalla, NY, USA) offers multiple models, including the EN-580 T (standard/therapeutic), EN-580XP (slim), and EI-580BT (short). These models range in outer diameters from 7.5 mm for the slim version to 9.4 mm for the standard and short versions, with working lengths of 155–200 cm and instrument channels ranging from 2.2–3.2 mm. Other models are also available. Like SBE, DBE systems require a balloon control unit for operation, and compatible overtubes are available in latex or silicone, with diameters tailored to the endoscope type (7, 41).

### Clinical applications

BAE has diverse applications in pediatrics, including both diagnostic and therapeutic uses. Indications include OGIB, ulcers, strictures, polyps, lymphangiectasia, chronic abdominal pain, chronic diarrhea, malabsorption syndromes, tumors, and eosinophilic enteritis, foreign body retrieval, post-small bowel transplantation evaluation, and jejunostomy tube placement (1, 2, 4, 5, 7, 12, 15, 16, 29, 41–48). In pediatric Crohn's disease, BAE facilitates targeted biopsies and therapeutic interventions like stricture dilation, reducing surgical interventions and guiding therapy (2, 5, 12, 29). For PJS, it enables safe and effective polypectomy, preventing complications like intussusception and bowel obstruction (46, 47).

Beyond its role in primary small bowel disorders, BAE is also useful in children with post-surgical or altered anatomy, where conventional endoscopy may be limited. It has been successfully used after procedures such as proctocolectomy, ileocecectomy, small bowel resection, serial transverse enteroplasty, and Roux-en-Y hepaticojejunostomy, with studies supporting its safety and efficacy (7, 45). One study reported that pediatric DBE can be safely performed postoperatively, though insertion challenges may arise due to adhesions, stenosis, or thickened bowel walls (45).

Diagnostic yields for SBE vary, with studies reporting rates of 47%–74% with no major adverse events (4, 5, 29, 41). DBE shows similar diagnostic yields, ranging from 58.8%–78.6% in pediatric cohorts, with therapeutic interventions in 46.5–76.9% of cases (1, 7, 15, 16, 48). Its ability to achieve deep intubation is advantageous for mid-to-distal small bowel pathologies, including polypectomy and stricture management (41, 47). Studies report SBE use in children ≥3 years and ≥13.5 kg (5, 42), and DBE in children ≥2 years and ≥12 kg (4, 15).

### Comparison of SBE and DBE

Although early studies suggested that DBE offered greater depth of insertion than SBE, more recent evidence indicates that the two techniques achieve comparable outcomes. A randomized multicenter trial by Domagk et al. reported mean oral intubation depths of 253 cm for DBE and 258 cm for SBE (49). Similarly, Efthymiou et al. found no significant difference between the two, with mean depths of 203.8 cm for SBE and 234.1 cm for DBE (36). A systematic review and meta-analysis by Lipka et al. confirmed these findings, showing no significant differences in anterograde or retrograde insertion depths between the two modalities (37). The choice between SBE and DBE is often determined by institutional availability, operator expertise, and the specific clinical indication, although some studies indicate DBE's dual balloons may provide enhanced therapeutic stability (50, 51).

### Safety and adverse events

BAE has a strong safety profile in pediatric patients, with low rates of major complications reported for both SBE and DBE, though outcomes may vary by patient population, procedural route, and clinical indication.

For SBE, multiple studies demonstrate excellent safety. Reddy et al. evaluated 189 SBE procedures in 174 children and reported no major adverse events, with minor issues such as transient abdominal pain being the most commonly observed complication (5). Similarly, Di Nardo et al. reviewed SBE use in children with Crohn's disease and reported no complications, further reinforcing the procedure's safety in this population (12). Among children with PJS, Bizzarri et al. reported mild abdominal pain in three cases and a single instance of post-polypectomy perforation out of 23 procedures. This perforation was managed conservatively without long-term complications, highlighting the relative safety of SBE for polypectomy in pediatric PJS patients (46). Li et al. also evaluated 41 children undergoing BAE for PJS and noted an overall complication rate of 1.2%, with 1.8% for BAE-facilitated polypectomy, supporting its safety in this high-risk population (14). Barth and Channabasappa similarly found no serious complications in their initial experience of SBE, concluding that SBE was both feasible and safe in children (52).

DBE shows a similarly favorable profile, though younger children may experience slightly higher minor complication rates. Yokoyama et al. reviewed 257 DBE procedures in 117 children, including DBE-cholangioscopy cases, and reported an overall complication rate of 5.4%, which increased to 10.4% in children under 10 years. Adverse events consisted of one case of post-polypectomy perforation and four cases of post-polypectomy bleeding (15). Chen et al. found no serious complications in 72 DBE procedures performed in 61 children, with minor issues like self-limited discomfort most common (48). Urs et al. reported complications in 5.2% of 113 DBE procedures in 58 children, all resolving without long-term sequelae (16). Lin and Erdman observed no major complications in 13 DBE procedures (43), and Nishimura et al. reported one case of post-polypectomy bleeding among 92 DBE procedures in pediatric patients (7).

A multicenter prospective study by Hagiwara et al. reviewed 96 procedures in pediatric patients and reported two severe adverse events: one case of bleeding after polypectomy and one instance of pancreatitis following retrograde DBE cholangioscopy. No intestinal perforations were observed, and the overall severe complication rate remained low (4).

While younger children, particularly those under 10 years, may experience slightly higher complication rates due to anatomical differences, BAE remains a safe and effective diagnostic and therapeutic tool in pediatric patients. In the available data, serious complications such as bleeding and perforation are rare and almost exclusively occur following polypectomy or other therapeutic interventions. Careful patient selection and procedural precision are essential to mitigate these risks.

### Training and implementation

Training and implementation of pediatric SBE and DBE require a structured educational framework and adherence to established clinical guidelines to ensure procedural safety and optimize patient outcomes. Competency is typically developed through a combination of clinical experience and simulation-based training, although no validated criteria currently exist for determining proficiency (53). Research has demonstrated that simulation-based training enhances technical skill acquisition and improves procedural performance in clinical settings (54–57). However, simulation alone cannot substitute for the hands-on clinical experience required to achieve expertise (58–60).

The ASGE recommends performing at least 10 upper deep enteroscopy cases and 20 retrograde DBE cases to achieve measurable improvements in DAE techniques, such as stable overtube intubation of the ileum. Meeting these minimum case volumes has been associated with greater procedural success, higher rates of complete small bowel examination, and shorter procedure durations (53). The ESGE advises completing at least 75 DAE procedures, including a minimum of 35 retrograde cases, with at least 50% of these involving therapeutic interventions, reflecting a more rigorous approach to competency (32).

Early studies have also highlighted the importance of case volume in building proficiency in DBE. One study demonstrated significant improvements in procedural and fluoroscopy times after 10 cases (61), while another suggested that full DBE expertise may require 100–150 cases (62).

### Research directions

BAE has been shown to be a safe and effective diagnostic and therapeutic tool for pediatric small bowel disorders. However, significantly fewer studies are available for SBE compared to DBE, particularly in pediatric populations. Larger multicenter trials are needed to better validate the safety and efficacy of SBE across diverse pediatric cohorts, which would also help refine techniques and protocols to minimize complications and improve patient safety (5, 12).

A critical research gap exists in understanding the comparative effectiveness of PE and BAE in pediatric patients. Although BAE offers greater insertion depth and therapeutic potential, comparative data in children is sparce. While SBE and DBE have shown comparable efficacy in adults, their effectiveness in pediatric populations remains underexplored. Studies are needed to evaluate SBE, DBE, PE, and capsule endoscopy in children, assessing diagnostic yield, therapeutic success, and complication profiles. Such studies would elucidate the advantages, limitations, and indications for each technique, optimizing procedural selection and clarifying the role of non-invasive imaging vs. the therapeutic potential of BAE and PE in pediatric gastroenterology.

Addressing these gaps also requires innovative approaches, including the adoption of emerging technologies to enhance current practices. In adult studies, artificial intelligence (AI) has demonstrated the potential to improve diagnostic accuracy and procedural efficiency, such as through enhanced lesion detection and reduced missed diagnoses (46, 63). Adapting AI for pediatric BAE could increase diagnostic yield, reduce the need for more invasive procedures, and ultimately enhance patient outcomes, helping to overcome some of the current limitations in this field.

## Spiral Enteroscopy (SE)

SE is an alternative technique for deep enteroscopy. The Endo-Ease Discovery SB system (Spirus Medical, Stoughton, Massachusetts, USA) uses a helical (spiral) overtube that rotates to pleat the small intestine, enabling deeper intubation. SE offers advantages such as reduced procedure time, easier setup compared to BAE, and a potentially shorter learning curve (31, 64–66). However, no pediatric-specific data exist, and its use in children is limited by the large 16 mm overtube diameter, which is unsuitable for smaller patients (6). Limited evidence and equipment availability currently hinder its adoption in pediatrics. Advances such as smaller-diameter overtubes are needed to evaluate its feasibility in pediatrics.

## Discussion

Deep enteroscopy has transformed the diagnostic and therapeutic landscape of pediatric small bowel disorders, providing solutions for conditions previously difficult to manage. Techniques like PE and BAE—encompassing SBE and DBE—have expanded the scope of minimally invasive endoscopy in pediatric gastroenterology. This review highlights advancements these techniques bring, while addressing challenges and future directions.

BAE has revolutionized small bowel diagnostics and therapeutic techniques, enabling interventions like polypectomy, stricture dilation, and hemostasis, reducing reliance on surgery, and improving outcomes. BAE's utility in managing OGIB, polyposis syndromes, and Crohn's disease is well-documented, with high diagnostic yields for SBE and DBE and strong safety profiles in pediatric cohorts.

Tailoring these techniques to pediatric populations remains challenging. Younger children often require procedural modifications due to their smaller size and distinct anatomy. Additionally, there is a scarcity of large-scale studies aimed at developing standardized protocols and conducting comparative evaluations of PE, SBE, DBE, and capsule endoscopy.

Training and competency development barriers include reliance on mentorship and adult-focused training that may not address pediatric-specific nuances. Structured pediatric training programs incorporating simulation could improve procedural skills and safety. Competency benchmarks must also be evaluated for their relevance to pediatric cases.

The future of pediatric deep enteroscopy lies in expanding its evidence base and integrating innovations like AI. Comparative studies are needed to refine procedural selection and tailor interventions. AI-driven diagnostic tools could enhance procedural efficiency and reduce operator dependency.

In conclusion, deep enteroscopy is indispensable for managing pediatric small bowel disorders. By addressing research gaps, refining training, and embracing innovations, its use in pediatrics can be further optimized, ensuring improved outcomes for children.
